# Experienced stressors and coping strategies among Iranian nursing students

**DOI:** 10.1186/1472-6955-6-11

**Published:** 2007-11-13

**Authors:** Naiemeh Seyedfatemi, Maryam Tafreshi, Hamid Hagani

**Affiliations:** 1 Mental Health Department, Faculty of Nursing and Midwifery affiliated to Iran Medical Sciences University, Yasemi, St. Valiasr Aven. Tehran, Iran; 2 Biostatistics Department, Faculty of Management and Information Science Center affiliated to Iran Medical Sciences University, Valiasr Ave. Rajaie Heart Center, Tehran, Iran

## Abstract

**Background:**

College students are prone to stress due to the transitional nature of college life. High levels of stress are believed to affect students' health and academic functions. If the stress is not dealt with effectively, feelings of loneliness, nervousness, sleeplessness and worrying may result. Effective coping strategies facilitate the return to a balanced state, reducing the negative effects of stress.

**Methods:**

This descriptive cross-sectional study was performed to determine sources of stress and coping strategies in nursing students studying at the Iran Faculty of Nursing & Midwifery. All undergraduate nursing students enrolled in years 1-4 during academic year 2004-2005 were included in this study, with a total of 366 questionnaires fully completed by the students. The Student Stress Survey and the Adolescent Coping Orientation for Problem Experiences Inventory (ACOPE) were used for data collection.

**Results:**

Most students reported "finding new friends" (76.2%), "working with people they did not know" (63.4%) as interpersonal sources of stress, "new responsibilities" (72.1%), "started college" (65.8%) as intrapersonal sources of stress more than others. The most frequent academic source of stress was "increased class workload" (66.9%) and the most frequent environmental sources of stress were being "placed in unfamiliar situations" (64.2%) and "waiting in long lines" (60.4%). Interpersonal and environmental sources of stress were reported more frequently than intrapersonal and academic sources. Mean interpersonal (P=0.04) and environmental (P=0.04) sources of stress were significantly greater in first year than in fourth year students. Among coping strategies in 12 areas, the family problem solving strategies, "trying to reason with parents and compromise" (73%) and "going along with family rules" (68%) were used "often or always" by most students.  To cope with engaging in demanding activity, students often or always used "trying to figure out how to deal with problems" (66.4%) and "trying to improve themselves" (64.5%). The self-reliance strategy, "trying to make their own decisions" (62%); the social support strategies, "apologizing to people"  (59.6%), "trying to help other people solve their problems" (56.3%), and "trying to keep up friendships or make new friends" (54.4%); the spiritual strategy, "praying" (65.8%); the seeking diversions strategy, "listening to music" (57.7%), the relaxing strategy "day dreaming" (52.5%), and the effort to "be close with someone cares about you"  (50.5%) were each used "often or always" by a majority of students.  Most students reported that the avoiding strategies "smoking" (93.7%) and  "drinking beer or wine" (92.9%), the ventilating strategies "saying mean things to people" and "swearing" (85.8%), the professional support strategies "getting professional counseling" (74.6%) and "talking to a teacher or counselor" (67.2%) and the humorous strategy "joking and keeping a sense of humor" (51.9%) were used "seldom or never".

**Conclusion:**

First year nursing students are exposed to a variety of stressors. Establishing a student support system during the first year and improving it throughout nursing school is necessary to equip nursing students with effective coping skills. Efforts should include counseling helpers and their teachers, strategies that can be called upon in these students' future nursing careers.

## Background

Stress has been identified as a 20th century disease and has been viewed as a complex and dynamic transaction between individuals and their environments [1]. Stressors can be broadly defined as situations or events that have the potential to affect health outcomes [2]. Stress can be regarded as a psychological threat, in which the individual perceives a situation as a potential threat [3].

Academic stress among college students has been a topic of interest for many years. College students, especially freshmen, are particularly prone to stress due to the transitional nature of college life. For example, many college students move away from home for the first time, which can necessitate leaving all previously learned support systems such as parents, siblings and high school friends. Students may need to develop entirely new social contacts and are expected to take responsibility for their own needs. They may have difficulty adjusting to more rigorous academic expectations and the need to learn to deal with individuals of differing cultures and beliefs. Thus, stress may result from being separated from home for the first time, the transition from a personal to an impersonal academic environment, and the very structure of the academic experience at the college level [4]. Significant changes in living conditions, the novel demands of the college academic environment, and the large change in social surroundings are just a few of the potential sources of stress for a college student [5]. College students experience high stress at predictable times each semester due to academic commitments, financial pressures, and lack of time management skills. Moreover, regardless of year in school, college students often deal with pressures related to finding a job or a potential life partner. These stressors do not cause anxiety or tension by themselves. Instead, stress results from the interaction between stressors and the individual's perception and reaction to those stressors. Other potential sources of stress for college students include excessive homework, unclear assignments, and uncomfortable classrooms. In addition to academic requirements, relations with faculty members and time pressures may also be sources of stress [6].

Stress-inducing academic demands include grade competition; lack of time and issues relating to time or task management, the need to adapt to new learning environments in terms of the increased complexity of the material to be learned and the greater time and effort required to do so; and the need to constantly self-regulate and to develop better thinking skills, including learning to use specific learning techniques. Another category that evokes stress is social adjustment, particularly adjusting to university life and separating from family and friends. Finally, there are financial pressures and other technical difficulties [7]. Archer and Lamnin (1985) found that tests, grades, competition, time demands, professors and the class environment, and concern about future careers were major sources of academic stress [8].

Stress and the identification of potential stressors among nursing students have received much attention in the literature [9]. Nursing students have the same academic stressors as other college students, such as midterm and final examinations, research papers and other assignments [1]. In addition, nursing students experience a clinical component, which is highly stressful. Students have a large amount of preparatory work before their clinical assignments. They often must travel long distances to clinical sites and use highly technical equipment [10,11]. In addition, they must perform procedures that can cause serious harm to their patients, thus enhancing their fear of making mistakes. Studies indicate that nursing students may be more prone to stress than other students. Beck and Srivastava (1991) performed a study to investigate the perception level and sources of stress across academic years in 94 nursing students enrolled in a baccalaureate nursing program at one university. Psychiatric symptoms were more prevalent in these students than in the general population. Many of the items ranked as stressful by the nursing students were also identified by other populations, such as amount of material to learn, examinations and lack of timely feedback from faculty. In addition, the nursing students identified feelings of inadequacy in dealing with acutely ill patients and difficulty in relationships with faculty. Nursing students had to devote long hours to study, were given multiple assignments, and lacked free time, timely feedback and faculty response to student needs[12].

In college and university students, some stress is motivating, whereas too high a level interferes with teaching [1]. Excessive stress can be harmful to a student's academic performance and students who perceive their stress levels as very high may often become depressed. This depression can lead to other mental health problems, such as excessive drinking or indiscriminate use of other substances [13]. Thus, academic stressors cover the whole area of learning and achieving, as well as adjusting to a new environment, in which a great deal of content must be assimilated in a seemingly inadequate period of time [7]. Moreover, excessive stress may lead a student to drop out of college [4]. If stress is not dealt with effectively, feelings of loneliness and nervousness, as well as sleeplessness and excessive worrying, may result. It is important that stress intervention programs be designed to address stress in college students. To design effective intervention programs, it is necessary to identify the stressors specific to college students.

Student perception of high stress levels can lead to poor academic performance, depression, attrition and serious health problems. Methods to reduce student stress often include effective time management, social support, positive reappraisal, and engagement in leisure pursuits [6]. Therefore, studying student stress and the methods students use to deal with it can have important implications for higher education administrators [11]. Although students cannot avoid these stressors, their ability to adjust to demands and cope with these stressors are important in achieving success in the college academic and social environments [5].

Coping has been viewed as a stabilizing factor that may assist individuals in maintaining psychosocial adaptation during stressful events. The process of coping is a very complex response that occurs when an individual attempts to remove stress or a perceived threat from the environment. Thus, the actual reaction to an environmental event may be as important as the event itself [13].

Coping responses can be described as positive or negative and as reactive (i.e. reacting to an individual's own thoughts and feelings) or active (dealing with actual stressful situations or events). Active or reactive coping responses can be positive or negative, depending on the situation and the content of the response [4].

The individual may deal with stress through several methods, including removing the stressor through manipulating the environment, developing specific responses to help deal with the stressor or seeking diversion from the stressor [13]. Researchers have found that ethnic, cultural and even socioeconomic characteristics influenced coping behaviors [7].

Failure to resolve student stress in the long term could have serious professional and personal consequences [9]. The primary objective of the present study was to identify the sources of stress in undergraduate nursing students. We also wished to assess whether there were any differences between students in different years of nursing school in their experience of stress sources and to determine the most and least common coping strategies used by these nursing students.

## Methods

The purpose of this descriptive cross-sectional study was to identify sources of stress in nursing students and to determine how they cope with stressful events. Research questions are:

What are the greatest stressors in different years?

What is the different between students of different years with regard to stressors?

What are the greatest coping mechanisms used by students?

The study population consisted of all 440 undergraduate nursing students enrolled in years 1–4 of an educational program at the Faculty of Nursing and Midwifery affiliated with Iran Medical Sciences University during the 2004–2005 academic year.

Written approval was obtained from the Ethical Review board of Iran Medical Sciences University, and permission was obtained from the Dean of Faculty, the Deputy of Educational Affairs and the Educational Affairs Administration to conduct the survey in the setting. After obtaining this approval for data collection, the researcher and her colleague introduced themselves to the students in each grade and informed all undergraduate students about the aim of this study and about guarantees of anonymity and confidentiality and the need for written informed consent. All students who agreed to participate and provided written informed consent were given questionnaires; all incomplete questionnaires were not included. A total of 366 students provided completed questionnaires.

Two survey instruments were used, The Student Stress Survey and the Adolescent Coping Orientation for Problem Experiences Inventory (ACOPE). The Student Stress Survey, based on the Student Stress Scale (Insel, & Roth, 1985), was used to measure stressors. This survey consists of 40 items divided into 4 categories of potential sources of stress: 6 items representing interpersonal sources of stress, 16 representing intrapersonal sources of stress, 8 representing academic sources of stress, and 10 representing environmental sources of stress. Interpersonal sources result from interactions with other people, such as a fight with a boyfriend or girlfriend or trouble with parents; intrapersonal sources result from internal sources, such as changes in eating or sleeping habits. Academic sources arise from school-related activities and issues, such as increased class workload or transferring between schools. Environmental sources result from problems in the environment outside of academics, such as car or computer problems and crowded traffic. Respondents provided a "Yes" or "No" answer to each item they had experienced during the current school year.

ACOPE has been used to assess coping strategies among adolescents, and was initially developed and tested with a sample of Midwestern junior and senior high school students by Patterson and McCubbin (1987). This scale has 54 items in 12 areas, each with Likert-scale responses (i.e. Never", "seldom", "Sometimes", "Often" and "Most of the time"): 6 items representing ventilating of feelings, 4 of relaxing, 8 of seeking diversions, 6 of developing self-reliance, 6 of optimism, 6 of developing social support, 6 of solving family problems, 5 of avoiding problems, 3 of seeking spiritual support, 2 of investing in close friends, 2 of seeking professional support, 4 of engaging in demanding activities, and 2 of being humorous.

After translating these 2 data gathering instruments in Farsi, their content validity was established by a panel of experts and their reliability was determined by Cronbach's Alpha, which was 0.78 for the Student Stress Survey and 0.85 for ACOPE.

All data were analyzed using SPSS for Windows Version 11. Sources of stress and coping strategies were assessed by descriptive analysis and groups were compared using inferential statistics. The binomial test was used to determine whether each source of stress was a significant stressor in all grades. Analysis of variance (ANOVA) was used to compare mean sources of stress in different years; for any that was significant, the Scheffe test was used to determine which group differed from the others.

## Results

Most of the students were females (87.2%) single (89.6%) and were between 18 and 24 years old. About 57% of the students lived in the university dormitories and came from cities other than Tehran, the capital city. In respect to answer the research questions: "What are the greatest stressors in different years?" and "What is the different between students of different years with regard to stressors"?" findings revealed that the most common interpersonal sources of stress were "finding new friends" (76.2%) and "working with people they did not know" (63.4%). The binomial test showed that "finding new friends" was a significant stressor in students of all years (P = 0.00). First year students found that "finding new friends", "working with people they did not know" and "change in social activities" were more frequently stressors than did other students; these differences were statistically significant (Tables 1, 2).

**Table 1:** Distribution of nursing students by experiencing interpersonal sources of stress

***Interpersonal Sources***	***Yes***		***NO***	
	
	*frequency*	*%*	*frequency*	*%*
finding new friend	279	76.2	87	23.8
Work with people they don't know	232	63.4	134	36.6
Roommate conflict	191	52.2	175	47.8
Change in social activities	176	48.1	190	51.9
Fight with boyfriend/girlfriend	114	31.1	252	68.9
Trouble with parents	74	20.2	292	79.8

**Table 2:** comparison of nursing students in different grade experiencing interpersonal sources of stress

***Interpersonal sources***	***year 1*** ***%***	***year 2*** ***%***	***year 3*** ***%***	***Year4*** ***%***
Change in social activities	65.4 P = 009	44.2	42.2	44
Roommate conflict	47.4	56.8	55.9	47.3
Work with people you don't know	80.7 P = 0.00	52.6	62.8	60.4
Finding New friend	94.9 P = 0.00	73.6 P = 0.00	73.5 P = 0.00	66 P = 003

The most common intrapersonal sources of stress were "new responsibilities" (72.1%) and "starting college"(65.8%). The binomial test showed that "new responsibilities" was a stressor in students of all years (p = 0.00). The factors "started college" and "change in sleeping habits" were significantly greater stressors in first year students than in students of other years (Tables 3, 4)

**Table 3:** Distribution of nursing students by experiencing intrapersonal sources of stress

***Interpersonal Sources***	***Yes***		***NO***	
	
	*frequency*	*%*	*frequency*	*%*
New responsibilities	264	72.1	102	27.9
Started college	241	65.8	125	34.2
Change in sleeping habits	202	55.2	164	44.8
Change in eating habits	194	53	172	47
Outstanding personal achievement	142	38.8	224	61.2
Financial difficulties	128	35	238	65
Spoke in public	101	27.6	265	72.4
Change in religious beliefs	87	23.8	279	76.2
Minor law violation	81	22.1	284	77.9
Decline in personal health	53	14.5	313	85.5
Held a job	50	13.7	316	86.3
Change in use of alcohol or drugs	48	13.1	318	86.9
Engagement/Marriage	43	11.7	323	88.3
Death of a family member	21	5.7	345	94.3
Death of a friend	21	5.7	345	94.3
Severe injury	18	4.9	348	95.1

**Table 4:** comparison of nursing students in different grades experiencing intrapersonal sources of stress

***Interpersonal sources***	***year 1*** %	***year 2*** %	***year 3*** %	***Year4*** %
Started college	91 P = 000	75.8 P = 000	55	32.9
Change in sleeping habits	75.6 P = 000	54.7	52	41.8
Change in eating habits	61.5	56.8	50	45
New responsibilities	89.7 P = 0.00	68.4 P = 0.00	63.8 P = 008	70 P = 0.00

The most common academic stressor was "increased class workload" (66.9%); the binomial test showed that this item was a significant stressor in students of all years (P = 0.00) (Tables 5, 6). Among environmental sources, most students reported that "being placed in unfamiliar situations" (64.2%) and "waiting in long lines" (60.4%) were the most frequent stressors. The binomial test showed that "being placed in unfamiliar situations" was a significant stressor in all 4 years (P = 0.00), whereas "waiting in long lines" and "change in living environment" were significantly greater in first year students than in students of other years (Tables 7, 8).

**Table 5:** Distribution of nursing students experiencing academic sources of stress

***Academic Sources***	***Yes***		***NO***	
	
	*frequency*	*%*	*frequency*	*%*
Increased class workload	245	66.9	121	33.1
Lower grade than anticipated	137	37.4	229	62.6
Anticipation of graduation	135	36.9	231	63.1
Search for graduate school/job	91	24.9	275	75.1
Missed too many classes	69	18.9	297	81.1
Serious argument with instructor	68	18.6	298	81.4
Change of Major	33	9	333	91
Transferred schools	18	4.9	348	95.1

**Table 6:** comparison of nursing students in different grades experiencing academic sources of stress

***Academic sources***	*year 1* %	*year 2* %	*year 3* %	*Year 4* %
Increased class workload	82 P = 000	73.7 P = 000	64.7 P = 000	49.5

**Table 7:** Distribution of nursing students experiencing environmental sources of stress

***Environmental sources***	***Yes***		***NO***	
	
	*frequency*	*%*	*frequency*	*%*
Placed in unfamiliar situation	235	64.2	131	35.8
Waited in long line	221	60.4	145	39.6
Car trouble	207	56.6	159	34.4
Vacations/breaks	197	53.8	169	46.2
Change in living environment	165	45.1	201	54.9
Messy living conditions	122	33.3	144	66.7
Computer problems	115	31.4	251	68.6
Put on hold for extended period of time	108	29.5	258	70.5
Quit job	9	2.5	357	97.5
Divorce between parents	1	0.3	365	99.7

**Table 8:** comparison of nursing students in different grades by experiencing environmental sources of stress

Environment sources	*year 1* %	*year 2* %	*year 3* %	*Year 4* %
Vacations/breaks	38.5	48.4	63.7	61.5
Waited in long line	67.9	64.2	59.8	50.5
Placed in unfamiliar situation	76.9 P = 000	84.6 P = 000	60.8 P = 0.003	51.6
Change in living environment	62.8	52.6	39.2	28.6
Car trouble	62.8	52.6	39.2	28.6

Mean stress was significantly greater in first year than in fourth year nursing students (36.4 vs 29.3, F = 3.39, P = 0.009). Using ANOVA, the mean of sources experienced in all 4 category differed significantly by year (F = 3.1, P = 0.04). The Scheffe test showed that the first year students had significantly higher means of interpersonal (3.33 vs. 2.68, P = 0.02) and environmental (4.02 vs. 3.15, P = 0.04) sources of stress compared with fourth year students. Moreover, third year students experienced significantly greater environmental stressors than fourth year students (4.03 vs. 3.15, P = 0.02).

In the area of ventilating feelings, most students reported seldom or never saying mean things to people, being sarcastic and "swearing" (85.5%), and some reported "crying" (33.3%) often or always (Table 9).

**Table 9:** Distribution of nursing students by using ventilating feelings

***Ventilating Feelings***	*Never/seldom*		*Sometimes*		*Often/always*	
	
	*No*	***%***	*No*	***%***	*No*	***%***
Say mean things to people, be sarcastic	314	85.8	34	9.3	18	4.9
Swear	314	85.8	33	9	19	5.2
Let off steam by complaining to your friends	211	57.7	118	32.2	69	18.9
Get angry and yell at people	209	57.1	90	24.6	67	18.3
Let off steam by complaining to family members	181	49.5	95	26	69	18.9
Cry	128	35	116	31.7	122	33.3

In the area of seeking diversions, the majority of students reported often or always "listening to music-stereo" (57.7%), while most reported "going to a movie" (71.9%) and "playing video games" (71.7%) seldom or never (Table 10).

**Table 10:** Distribution of nursing students by seeking diversions

***Seeking Diversions***	*Never/seldom*		*Sometimes*		*Often/always*	
	
	No	**%**	No	**%**	No	**%**
Go to a movie	263	71.9	70	19.1	33	9
Play video games	262	71.7	61	16.7	43	11.7
Use drugs	233	64.5	55	15	75	20.5
Listen to music-stereo	63	17.2	92	25.1	211	57.7
Read	110	30.1	134	36.6	122	33.3
Go shopping, buy things you like	156	42.6	111	30.3	99	27
Watch T. V	114	31.1	105	28.7	147	40.2
Sleep	113	30.9	118	33.2	135	36.9

In the area of relaxing, the majority reported "daydreaming" (52.5%) often or always, while most reported "working on a hobby" (85.5%) seldom or never (Table 11).

**Table 11:** Distribution of nursing students by Relaxing

***Relaxing***	*Never/seldom*		*sometimes*		*Often/always*	
	
	No	**%**	No	**%**	No	**%**
Work on a hobby	214	85.5	73	19.9	79	216
Eat food	188	51.4	105	28.7	73	19.9
Daydream	80	21.9	94	25.8	192	52.5
Ride around in the car	238	65	75	20.5	53	14.5

In the area of self-reliance, the majority reported "trying to make their own decisions" (62%), and "trying to think of good things" (50.8%) "often or always", while most reported "getting a job or working harder" (80.3%) seldom or never (Table 12).

**Table 12:** Distribution of nursing students by self-Reliance

***Self-Reliance***	*Never/seldom*		*Sometimes*		*Often/always*	
	
	*No*	***%***	*No*	***%***	*No*	***%***
Get a job or work harder	294	80.3	53	14.5	19	5.2
Try to think of the good things	68	18.6	112	30.6	186	50.8
Try to make your own decisions	52	14.2	87	23.8	227	62
Organize your life and what you have to do	95	26	91	24.9	180	49.2
Get more involved in activities in school	87	23.8	137	37.4	142	38.8
Tell yourself the problem(s) is not important	124	33.9	105	28.7	137	37.4

In the area of developing social support, the majority reported "apologizing to people" (59.6%), "trying to help other people solve their problems" (56.3%) and "trying to keep up friendships or make new friends" (54.4%) often or always, whereas most said that they seldom or never "blamed others for what is going on" (62.6%) (Table 13).

**Table 13:** Distribution of nursing students by Developing Social Support

***Developing Social Support***	*Never/seldom*		*Sometimes*		*Often/always*	
	
	*No*	***%***	*No*	***%***	*No*	***%***
Blame others for what's going on	229	62.6	90	24.6	47	12.8
Apologize to people	39	13.4	99	27	218	59.6
Try to help other people solve their problems	63	17.2	97	26.5	206	56.3
Try to keep up friendships or make new friends	72	19.7	95	26	199	54.4
Say nice things to others	126	34.4	122	33.3	118	32.2
Talk to a friend about how you feel	125	34.2	105	28.7	136	37.2

In the area of solving family problems, most students reported often or always "trying to reason with parents and talk things out, compromise" (73%) and "going along with parent's requests and rules" (68%), while most said they seldom or never "talked to their own fathers about what bothers them" (60.4%) (Table 14).

**Table 14:** Distribution of nursing students by Solving Family Problems

***Solving Family Problems***	*Never/seldom*		*Sometimes*		*Often/always*	
	
	*No*	***%***	*No*	***%***	*No*	***%***
Talk to your father about what bothers you	221	60.4	68	18.6	77	21
Try to reason with parents and talk things out, compromise	34	9.2	65	17.8	267	73
Go along with parent's requests and rules	35	9.6	82	22.4	249	68
Do things with your family	105	28.7	108	29.5	153	41.8
Talk to a brother or sister about how you feel	152	41.5	102	27.9	112	30.6
Talk to your mother about what bothers you	97	26.5	100	27.3	169	46.2

In the area of avoiding, almost all students said that smoking (93.7%), using drugs prescribed by a doctor (79%), and trying to stay away from home (66.1%) were used often or always, whereas 43.2% reported seldom or never "trying to see the good things" (Table 15).

**Table 15:** Distribution of nursing students by Avoiding

***Avoiding***	*Never/seldom*		*Sometimes*		*Often/always*	
	
	*No*	*%*	*No*	*%*	*No*	*%*
Smoke	343	93.7	4	1.1	19	5.2
Use drugs prescribed by doctor	289	79	15	13.9	26	7.1
Try to stay away from home as much as possible	242	66.1	78	21.3	46	12.6
Try to see the good things in a difficult situation	103	28.1	105	28.7	158	43.2
Drink beer, wine, liquor	340	92.9	11	3	15	4.1

In the area of seeking spiritual support, the majority reported often or always "praying" (65.8%), while most said that "talking to a minister/priest/rabbi" (68%) and "going to a mosque" (55.2%) were used seldom or never (Table 16).

**Table 16:** Distribution of nursing students by Seeking Spiritual Support

***Seeking Spiritual Support***	*Never/seldom*		*Sometimes*		*Often/always*	
	
	*No*	*%*	*No*	*%*	*No*	*%*
Talk to a minister/priest/rabbi	249	68	80	21.9	37	10.1
Go to mosque	202	55.2	95	26	69	18.9
Pray	53	14.5	72	19.7	241	65.8

In the area of investing in close friends, a majority reported that "being close with someone you care about" (50.5%) was used often or always (Table 17).

**Table 17:** Distribution of nursing students by Investing in Close Friends

***Investing in Close Friends***	*Never/seldom*		*Sometimes*		*Often/always*	
	
	*No*	*%*	*No*	*%*	*No*	*%*
Be close with someone you care about	86	23.5	95	26	185	50.5
Be with a boy friend or girlfriend	123	33.6	113	30.9	130	35.5

In the area of seeking professional support, the majority reported that "getting professional counseling" (74.6%) and "talking to a teacher or counselor at school about what bothers them" (67.2%) were used seldom or never (Table 18).

**Table 18:** Distribution of nursing students by Seeking Professional Support

***Seeking Professional Support***	*Never/seldom*		*Sometimes*		*Often/always*	
	
	*No*	*%*	*No*	*%*	*No*	*%*
Get professional counselling	273	74.6	54	14.8	39	10.7
Talk to a teacher or counsellor at school about what bothers you	246	67.2	72	19.7	48	13.1

In the area of engaging in demanding activity, most students reported that "trying, on their own, to figure out how to deal with problems or tension" (66.4%) and "trying to improve oneself" (64.5%) were used often or always, while "performing strenuous physical activity" (77.3%) was used seldom or never (Table 19).

**Table 19:** Distribution of nursing students by Engaging in Demanding Activity

***Engaging in Demanding Activity***	*Never/seldom*		*Sometimes*		*Often/always*	
	
	*No*	*%*	*No*	*%*	*No*	*%*
Do a strenuous physical activity (jogging, biking, etc	283	77.3	55	15	28	7.7
Try, on your own, to figure out how to deal with your problems or tension	46	12.6	77	21	243	66.4
Try to improve yourself (get body in shape, get better grades, etc.)	59	16.1	71	19.4	236	64.5
Work hard on school work or school projects	203	55.5	94	25.7	69	18.9

In the area of being humorous, a majority reported that "joking and keeping a sense of humor" (51.9%) was used seldom or never (Table 20).

**Table 20:** Distribution of nursing students by Being Humorous

***Being Humorous***	*Never/seldom*		*Sometimes*		*Often/always*	
	
	*No*	*%*	*No*	*%*	*No*	*%*
Joke and keep a sense of humor	190	51.9	93	25.4	83	22.7
Try to be funny and make light of it all	100	28.7	105	28.7	156	42.4

## Discussion

The aim of this study was to assess the sources of stress among nursing students and the coping strategies they used to overcome these stresses. We found that interpersonal and environmental sources of stress were the most common, with the five most frequently reported stressors being finding new friends, new responsibilities, increased class workload, being placed in unfamiliar situations, and working with people they did not know, in that order. Most of these undergraduate students come from townships outside Tehran and study for 4 years in that city. They are therefore placed in unfamiliar surroundings, with crowded streets and other stressors endemic to a capital city. Thus, for these students, living in dormitories may cause more stress than for students who live in Tehran. In addition, this data was collected during the Fall Semester, when students are at the beginning of their courses, have the responsibility to do homework, have to start attending new clinical units for training.

During their college years, students experience constant challenges and demands for adjustment and change. Along with academic pressures, students must take responsibility for themselves, must seek acceptance from their peers in a world of mixed values, and begin more intimate relationships. Mahat (1998) found that negative interpersonal relationships were the most frequently reported stressful event [10], while Ross (1999) found that intrapersonal sources of stress were the most common [14]. Among our cohort of students, the four least frequently reported stressors were divorce between parents, quitting a job, severe injury, and transferring schools, in agreement with Ross (1999). In comparison, Evans & Kelly (2004) found that examinations, the intense amount of work, and finding the academic work difficult were the most important source of stress. Nigerian nursing students have high levels of stress, with the most common stressors including excessive schoolwork, financial problems, inadequate recreational facilities, and overcrowded accommodations [15]. These findings indicated a need for counseling and other support services among nursing students.

We found that first-year students experienced greater stress than students in subsequent years, findings in agreement with those reported previously (Walton, 2000). In addition, Misra & McKeen (2000) found that students at the freshman and sophomore levels experienced more stress than juniors or seniors.

WHO/EHA guidelines have stated that there are no standards for coping strategies; rather, they vary depending on socio-cultural factors. Coping strategies have been shown to vary by region, community, social group, household, gender, age, season and time in history and are greatly influenced by an individual's previous experiences [16]. We found that the most commonly used coping strategies are, in order: going along with one's parents requests and rules, praying, making one's own decisions, apologizing, helping other people to solve problems, keeping friendships and daydreaming. In Iran, the family is important, both culturally and religiously, with religiosity being the most striking cultural feature in the country. Thus, respecting one's family's rules, parents' requests and praying are emphasized in childrearing. Every Muslim learns prayer from childhood and prays five times daily. In addition, Iranians are very social and emotional people, so that helping each other, maintaining friendships and complimentary rituals such as apologizing are prominent attributes of Iranians. Among Black and Latino students, the most frequently reported coping strategies were talking with friends, talking with parents, and participating in religious and social activities [17]. In addition, a majority of students were found to utilize the "seeking social support" category of coping more than others [10], a finding supported by this study. Among pre-registration diploma nursing students in Ireland, talking to relatives and friends and talking to peers were the most common methods of coping with stress [1].

We found that the least common coping strategies used by nursing students were, in order, smoking, drinking, using prescribed drugs, getting professional counseling, going to movies, playing video games, talking to a counselor, talking to a priest, and staying away from home. Although many studies have found high smoking and drinking rates among students, these rates are low in Iranian students [18-23]. This may be related to the religious beliefs of students, which prohibit such behaviors. While talking to a counselor and getting professional counseling are cultural behaviors, many people may refuse professional help, except for severe problems. In contrast, 24.6% of Black and Latino students indicated that they would talk to a counselor about their concerns [17].

## Conclusion

It is clear from the results of this study that the Iranian student nurses surveyed, especially freshmen, were exposed to a variety of interpersonal and environmental stressors. These findings indicate the need for stress management programs specific to the needs of college students. Given the detrimental effects of stress on health and academic performance, college administrators should consider incorporating stress management training into orientation activities for nursing students [14]. At a minimum, the most commonly identified sources of stress should be discussed with incoming freshmen. Furthermore, students should be informed of the campus resources available to help them address these stresses. One approach may be the use of a stress management workshop, specifically geared to the stressors encountered by college students. Educational administrators should introduce effective coping strategies through counseling programs for newcomers and they should support at risk students during their studies. Orientation programs for first-year students should include stress management as a topic of discussion, workshops on stress and strategies to cope with stress. Such workshops might also be conducted during the academic year. The presence of a counseling team among the faculty is necessary.

### Study Limitations

One important limitation of this study was that we used a small sample of students, drawn from just one of the nursing faculties in Tehran. Our findings cannot be generalized for students in other degree programs, such as those in master's or doctoral degree programs. The self-report questionnaire used also carried the risk that respondents would answer in a socially desirable manner. Repeat of this study with a larger, randomized sample would expand knowledge of stress among nursing students.

## Competing interests

The author(s) declare that they have no competing interests.

## Authors' contributions

N S Corresponding author, participated in the design of the study, gathering data and performed the statistical analysis, prepared study report and its translation

M T participated in data gathering and in the sequence alignment and drafted the manuscript,

H H participated in statistical analysis and preparing discussion section

All authors read and approved the final manuscript

## Pre-publication history

The pre-publication history for this paper can be accessed here:



## References

[B1] Evans W, Kelly B (2004). Pre-registration diploma student nurse stress and coping measures. Nurse Education Today.

[B2] Barling J (1990). Employment, stress and family functioning.

[B3] Day AL, Livingstone HA (2004). Gender differences in perceptions of stressors and utilization of social support among university students. Canadian Journal of Behavioral Science.

[B4] Shields N (2001). Stress, active coping, and academic performance among persisting and nonpersisting students. Journal of Applied Biobehavioral Research.

[B5] Tamina Toray (1998). Coping in Women College Students: The Influence of Experience.

[B6] Misra R, McKean M (2000). College students' academic stress and its relation to their anxiety, time management, and leisure satisfaction. American Journal of Health Studies.

[B7] Kariv D, Heiman T (2005). Task-oriented versus emotion-oriented coping strategies: the case of college students. College Student Journal.

[B8] Archer J, Lamnin A (1985). An investigation of personal and academic stressors on college campuses. Journal of College Student Personnel.

[B9] Nicholl H, Timmins F (2005). Programme-related stressors among part-time undergraduate nursing students. Journal of Advanced Nursing.

[B10] Mahat G (1998). Stress and coping: junior baccalaureate nursing students in clinical settings. Nursing Forum.

[B11] Shriver CB, Scott-Stiles A (2000). Health habits of nursing versus non-nursing students: a longitudinal study. Journal of Nursing Education.

[B12] Beck DL, Srivastava R (1991). Perceived level and sources of stress in baccalaureate nursing students. Journal of Nursing Education.

[B13] Robin WaltonL (2002). A Comparison of Perceived Stress Levels and Coping Styles of Junior and Senior Students in Nursing and Social Work Programs. Doctor of Education Dissertation.

[B14] Ross SE, Niebling BC, Heckert TM (1999). Sources of stress among college students. College Student Journal.

[B15] Omigbodun OO, Onibokun AC, Yusuf BO, Odukogbe AA, Omigbodun AO (2004). Stressors and counseling needs of undergraduate nursing students in Ibadan, Nigeria. Journal of Nursing Education.

[B16] WHO/EHA (1999). Overview Coping Mechanisms. Emergency health training programme for Africa.

[B17] Chiang L, Hunter CD, Yeh CJ (2004). Coping attitudes, sources, and practices among Black and Latino college students. Adolescence.

[B18] Gostautas A, Povilaitis R, Pilkauskiene I, Jakusovaite I, Statkeviciene S (2007). The peculiarities of use of addictive substances among students during 2005–2006. Medicina (Kaunas).

[B19] Fox K (2006). Alcohol and drug use among dental and law undergraduates. British Dental Journal.

[B20] Smith DR, Leggat PA (2007). Tobacco smoking habits among a complete cross-section of Australian nursing students. Nursing & Health Sciences.

[B21] Karam E, Kypri K, Salamoun M (2007). Alcohol use among college students: an international perspective. Current Opinion in Psychiatry.

[B22] Barber MW, Fairclough A (2006). comparison of alcohol and drug use among dental undergraduates and a group of non-medical, professional undergraduates. British Dental Journal.

[B23] Watson H, Whyte R, Schartau E, Jamieson E (2006). Survey of student nurses and midwives: smoking and alcohol use. British Journal of Nursing.

